# A comprehensive retrospective study of the seroprevalence of H9N2 avian influenza viruses in occupationally exposed populations in China

**DOI:** 10.1371/journal.pone.0178328

**Published:** 2017-06-02

**Authors:** Xin Li, Bai Tian, Zhou Jianfang, Chen Yongkun, Li Xiaodan, Zhu Wenfei, Li Yan, Tang Jing, Guo Junfeng, Chen Tao, Gao Rongbao, Wang Dayan, Yuelong Shu

**Affiliations:** National Institute for Viral Disease Control and Prevention, Chinese Center for Disease Control and Prevention, Key Laboratory for Medical Virology, Ministry of Health, Beijing, People’s Republic of China; University of Hong Kong, HONG KONG

## Abstract

The H9N2 avian influenza virus circulates worldwide, predominantly in poultry. Its increasing infectivity and adaptation in poultry and mammals have enhanced the possibility of human infection. However, H9N2 human cases are difficult to detect due to their mild clinical symptoms. Serological study is valuable for risk assessment. A total of 15,700 serum samples were collected from occupationally exposed populations in 22 provinces of China and tested using hemagglutination inhibition (HI) and microneutralization (MN) assays. The sera positive rate of A/Guangzhou/333/99 (G9) was significantly higher than that of A/quail/Hong Kong/G1/97 (G1) (*p*<0.0001). The seroprevalences of H9N2 were significantly higher in live poultry market workers, large-scale poultry farmers and backyard farmers than in poultry slaughtering factory workers and wild bird habitant workers. The seroprevalences of A/Guangzhou/333/99 (G9) (3.42%) and A/quail/Hong Kong/G1/97 (G1) (1.37%) in Southern China were significantly higher than those in Northern China (*p*<0.001). The seroprevalence was highest in the elderly, followed by adults and then youths. Our results indicate that subclinical human infection with H9N2 avian influenza virus is widely distributed in China. Longer poultry exposure might contribute to the higher seroprevalence in the elderly group. The higher seroprevalence observed in Southern China than in Northern China might be caused by a higher poultry density.

## Introduction

In China, lowly pathogenic H9N2 avian influenza viruses are the most prevalent avian influenza viruses in poultry [1, 2]. This strain was first isolated from a turkey in the United States in 1966 [3], and it subsequently spread to Asia, Europe, the Middle East and Africa [1]. As indicated by the hemagglutination (HA) gene sequences reported in the influenza virus resource database of the US National Center of Biotechnology Information (NCBI), more than 90% of the H9N2 viruses isolated worldwide are from Asia, with approximately 78% of them from China [4, 5]. H9N2 can be divided into four lineages based on phylogenetic tree analysis: G1, G9 (or Y280), Korea (Y439) and North America [5]. G9 and G1 are the predominate lineages in China and have been isolated from all parts of the country over the past few years [1].

Since the first human infection with H9N2 virus was reported in 1998 [6], concern for its pandemic potential has been increasing, especially in recent years. Surveillance data showed that the incidence of the Q226L substitution in the H9N2 HA gene exceeded 75% of the H9N2 viruses that are currently in mainland China [1]. This receptor-binding site substitution enhanced virus transmission among ferrets in experimental conditions [7] and the possibility of human infection. Host ranges of the H9N2 virus have expanded to pigs [8], dogs and cats [9]. A recent study showed that H9N2 had increased infectivity in chickens [10]. In addition, H9N2 exhibits a high gene compatibility with other influenza A viruses. H9N2 virus donated its six internal genes to H5N1 [11], H7N9 [12] and H10N8 [13] viruses, thus transferring over to humans naturally. In experimental conditions, H9N2 virus re-sorted with pandemic H1N1 [14], H5N1 [15] or H3N2 [16] viruses, resulting in enhanced pathogenicity and transmissibility in mammals. With the increasing threat posed by H9N2 viruses, evaluating their public health risk is important.

The clinical symptoms of H9N2 human infection are always mild [17], which makes it difficult to find human cases through regular surveillance systems. A serological survey is an optimal approach for identifying subclinical cases of infections and for investigating the risk of transmission to humans. In recent years, a number of serology studies were performed in Guang Zhou [18], Jiang Su [19], Shan Dong [20], Shang Hai [21] and Beijing [22] in China. The seroprevalence of H9N2 varied between different areas and different populations. No single study created a panoramic view of the seroprevalence of H9N2 in China. Since 2009, China has implemented a serology surveillance system among occupationally exposed populations to monitor and to evaluate the risk of human infection with the highly pathogenic avian influenza H5N1 virus. In the current study, a systematic and comprehensive seroprevalence study of H9N2 avian influenza viruses was performed using the serum samples collected by the surveillance system for H5N1.

## Methods

### Virus and ferret antisera

Based on avian influenza surveillance data from China, 2 representative H9N2 viruses (A/Guangzhou/333/99 (G9) and A/quail/Hong Kong/G1/97 (G1), a representative virus of the G1 lineage) were selected as the antigens for antibody detection. A/Guangzhou/333/99 (G9) was isolated from a human case by the Chinese National Influenza Center, and A/quail/Hong Kong/G1/97 (G1) was kindly provided by Dr. Jin-hua Liu. Ferret antisera raised against these viruses were used as positive controls for the HI and MN assays with the approval of the Animal Care Welfare Committee of the National Institute for Viral Disease Control and Prevention, Chinese Centers for Disease Control and Prevention.

### Serum samples

Since 2008, China has implemented a routine national serologic surveillance of occupationally exposed populations for the highly pathogenic avian influenza H5N1 virus. As part of the national pandemic-preparedness plan, the requirement for informed consent was waived according to the regulatory policy in China. In this study, a total of 15,700 stored serum samples that had been collected between 2009 and 2011 were used. Poultry workers from live poultry markets, large-scale poultry farms, backyard poultry farms, poultry slaughter factories and wild bird habitats were included in this study. Detailed demographic information is shown in Table 1. The serum analysis in this study included 13236 sera that were tested in parallel against both A/Guangzhou/333/99 and A/quail/Hong Kong/G1/97 antigens, and 2464 sera that were tested for only one of the antigens due to inadequate sera.

**Table 1:** Antigenic analysis of H9N2 G9 lineage viruses.

	Ferret antisera				
Antigen	333	AK4	AE15	GB26	NX184
A/Guangzhou/333/99	**640**	320	160	80	20
A/Chicken/Anhui/AK4/2011*	640	**1280**	640	320	320
A/Chicken/Anhui/AE15/2011*	640	640	**640**	320	160
A/Chicken/Guizhou/GB26/2011#	320	640	320	**640**	320
A/Chicken/Ningxia/NX184/2011#	160	320	40	320	**640**
A/Environment/jiangxi/21/2011	640	1280	640	640	320
A/Environment/Guangdong/01269/2012	320	1280	640	1280	640

Viruses marked * and # were generously donated by Dr. Jiming Chen, whereas viruses marked * were the predominate lineage h9.4.2.5 circulating in China until 2011, and viruses marked # were the newly emerging lineage h9.4.2.6 in 2011 [1]. In addition, two environment H9N2 virus isolates and one old H9N2 virus, A/Guangzhou/333/99, isolated from human in 1999 were selected. Virus abbreviations: 333, A/Guangzhou/333/99; AK4, A/Chicken/Anhui/AK4/2011; AE15, A/Chicken/Anhui/AE15/2011; GB26, A/Chicken/Guizhou/GB26/2011; NX184, A/Chicken/Ningxia/NX184/2011. The HI test was performed using 1% turkey red blood cells.

### Hemagglutination inhibition (HI) assay

For the HI assay, all serum samples were treated with 4 volumes of receptor-destroying enzyme (RDE, prepared by the Chinese National Influenza Center) at 37°C for 18 hours, followed by inactivation at 56°C for 30 min. All serum samples were then adsorbed by packed turkey red blood cells (one volume of packed red blood cells to 10 volumes of RDE-treated sera) for 60 min at 4°C to remove nonspecific hemagglutinin. The HI assay was performed as previously described using 1% turkey red blood cells. Serum samples were titrated in 2-fold dilutions in phosphate-buffered saline and tested at an initial dilution of 1:10. Viruses were inactivated using 0.5‰ β-propiolactone. A Beckman automatic workstation (BioMek FXP, USA) was used to dilute the sera and to add the antigen and red blood cells. Individuals with an HI titer of ≥40 were considered suspected seropositive, and the samples were then confirmed by MN assay.

### Microneutralization (MN) assay

MN assay was performed according to the WHO Manual for the Laboratory Diagnosis and Virological Surveillance of Influenza [23]. Human sera were inactivated at 56°C for 30 min, and ferret sera were treated by RDE. Briefly, the sera were serially diluted two-fold and then pre-incubated with 50 μl of 100 TCID_50_ of the virus in MEM containing 1% BSA for 1 h before 100 μl of 1.5 x 10^5^ MDCK cells/ml were added. After 18 h of incubation at 37°C, the cells were fixed by 80% acetone, and the influenza A virus NP protein in infected cells was detected by ELISA using a mixture of two nucleoprotein (NP) primary antibodies (Millipore, MAB8257 and MAB8258). The titer was calculated using the Reed-Muench method [24]. An MN titer of ≥40 was selected as the endpoint.

### Statistical analysis

Seroprevalence was analyzed by chi-square or Fisher's exact tests using GraphPad Prism5 (La Jolla, CA, USA) software. *P*<0.05 was used to indicate a significant difference.

## Results and discussion

### Seroprevalence of H9N2 viruses

In a preliminary study, a total of 8 representative H9N2 viruses (one of G1 lineage and seven of G9 lineage) that genetically belonged to different lineages or sublineages were antigenically analyzed by HI assay using ferret antisera raised against these viruses. The results showed that the G1 and G9 lineage viruses were antigenically significantly different. For G9 lineage viruses, a study published in 2012 reported that two G9 lineages, h9.4.2.5 and h9.4.2.6, were circulating in mainland China before 2012, with h9.4.2.5 being the predominate lineage and h9.4.2.6 a newly emerging lineage [1]. Hence, A/Guangzhou/333/99 (G9 lineage), which is antigenically similar to h9.4.2.5 lineage viruses, was selected as a representative (Table 1). In addition, A/quail/Hong Kong/G1/97 (G1 lineage) was selected as a representative of the G1 lineage virus. In this serology study, an HI assay was performed for all of the serum samples. A total of 1912 samples against A/Guangzhou/333/99 (G9) and 489 samples against A/quail/Hong Kong/G1/97 (G1) with HI≥40 were selected for verification using the MN test. An MN antibody titer greater than 40 was considered positive for this seroprevalence analysis (Table 2). Overall, the seroprevalence of A/Guangzhou/333/99 (G9) was significantly higher than that of A/quail/Hong Kong/G1/97 (G1) (3.04% VS 1.18%, *p*<0.0001).

**Table 2:** Seroprevalence of occupationally exposed populations against H9N2 viruses by MN assay.

Characteristics	A/Guangzhou/333/99				A/quail/Hong Kong/G1/97			
	Tested sera *	MN≥40	Seroprevalence (%)	95%CI #	Tested sera *	MN≥40	Seroprevalence(%)	95%CI #
**Occupation**								
Overall population	14896	453	3.04	2.99–3.09	13453	159	1.18	1.16–1.20
Live poultry market	4021	156	3.88	3.76–4.00	3480	38	1.09	1.05–1.13
Large-scale poultry farm	3872	115	2.97	2.88–3.06	3463	57	1.65	1.60–1,70
Backyard poultry farm	4121	130	3.15	3.06–3.25	3985	52	1.3	1.26–1.34
Poultry slaughter factory	1228	26	2.12	2.00–2.23	970	5	0.52	0.49–0.55
Wild bird habitat	664	11	1.66	1.53–1.78	565	5	0.88	0.81–0.95
Others	990	15	1.52	1.42–1.61	990	2	0.19	0.18–0.20
**Gender**								
Female	7211	204	2.83	2.76–2.89	6462	68	1.05	1.02–1.08
Male	7685	249	3.24	3.17–3.31	6991	91	1.3	1.27–1.33
**Age groups**								
Children (-14)	75	0	0	-	65	1	1.54	1.17–1.91
Youth(15–24)	1157	16	1.38	1.30–1.46	1007	4	0.39	0.37–0.41
Adult(25–59)	11865	363	3.06	3.01–3.11	10666	118	1.1	1.08–1.12
Elderly(60-)	1640	73	4.45	4.24–4.66	1474	34	2.31	2.19–2.43
No age record	159	1	0.62	-	241	2	-	-

* Total number of collected sera samples was 15,700, of which 13,236 sera were tested in parallel against both A/Guangzhou/333/99 (G9) and A/quail/Hong Kong/G1/97 (G1) antigens and 2464 sera were tested for only one of the antigens due to inadequate sera.

# 95%CI: 95% confidence interval. It was calculated by SPSS (17.0, Armonk, NY, USA).

The sera tested are not contemporary; thus, he manuscript cannot provide the true picture of how much H9N2 infection is currently in the Chinese population. The sera tested were from occupationally exposed populations that may not be a representative sample of the Chinese population; as a result, the generalizability of the study results is limited. As it is reported, the seroprevalence of the H9N2 virus was significantly higher than that of H5N1 in populations in China [18]. This result indicates that the H9N2 virus could infect humans more easily than H5N1 because the receptor binding preference of the H9N2 virus is for human-like receptors [1]. Only approximately 30 human cases of H9N2 have been reported since 1998; however, 453 subjects presented H9N2 neutralization antibodies, which suggests more human infections occurred than were reported. Since the study subjects were occupationally exposed populations, they were mostly from the countryside and had poor access to medical care. Infected subjects with mild clinical symptoms or subclinical infection were difficult for the influenza surveillance system to identify. This is the first time that serology investigation was conducted for H9N2 viruses across China. Previously, several studies reported the seropositivity rates of H9N2 viruses in small specific regions in China. The seropositivity rates in occupationally exposed populations, using the MN method, were 4.6% in Beijing [22] and 2.3% in Shandong province [20], which were similar to our result. In some regions of China, the seroprevalence in poultry exposure populations is much higher, as seen with a rate of 15.5% in poultry retailers in Guangdong province [18]. In the general population (non-poultry-exposed populations), the seroprevalences were decreased to 1.3% in Guangzhou [18] and 1.4% in Jiangsu province [19].

### Seroprevalence of H9N2 viruses among different occupations

**Fig 1:**
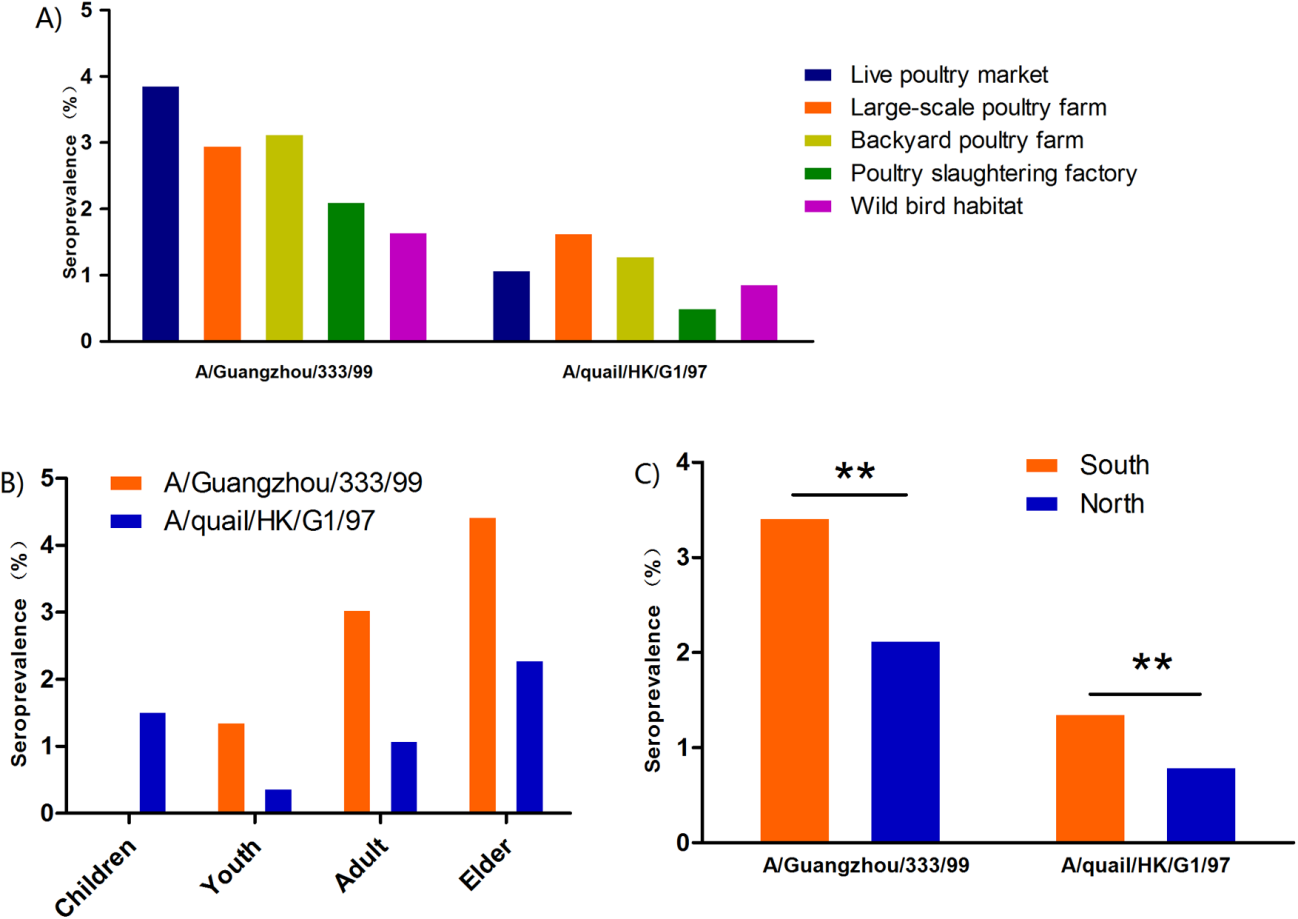
Comparison of seroprevalence from different human populations and regions in China. A) Comparison of the seropositivity of H9N2 viruses in occupationally exposed populations. The seropositivities among different occupationally exposed populations were significantly different for H9N2 viruses (*p* = 0.0023 for A/Guangzhou/333/99 (G9) and *p* = 0.041 for A/quail/Hong Kong/G1/97(G1)). B) Comparison of the seroprevalence of H9N2 viruses in different age groups. Children: aged up to 14; Youth: aged 15–24; Adult: aged 25–59; Elderly: aged over 60. The seroprevalences of H9N2 viruses in different age groups were significantly different (*p*<0.0001 for both H9N2 viruses). C) Comparison of the seroprevalence of H9N2 antigens in Northern and Southern China. ** indicates *p*≤0.001.

The seroprevalence of H9N2 viruses was calculated and compared between different occupational groups. The seroprevalences of A/Guangzhou/333/99 (G9) were markedly higher than those of A/quail/Hong Kong/G1/97 (G1) among different occupationally exposed populations. For A/Guangzhou/333/99 (G9), the seroprevalences among different occupationally exposed populations were significantly different (*p* = 0.0023). The seroprevalence in live poultry market workers was 3.88%, which was the highest among all occupationally exposed populations (Fig 1A). The seroprevalences in large-scale poultry farmers (2.97%) and backyard poultry farmers (3.15%) were lower than those in live poultry market workers but higher than those in poultry slaughtering factory workers (2.12%) and wild bird habitat workers (1.66%) (Fig 1A). For A/quail/Hong Kong/G1/97 (G1), the positive rates of neutralization antibodies in occupationally exposed populations were significantly different (*p* = 0.041). The large-scale poultry farmers (1.65%), backyard farmers (1.3%) and live poultry market workers (1.09%) were the top three groups that had positive rates over 1% (Fig 1A). The seropositivity rates of poultry slaughtering factory workers and wild bird habitat workers were 0.52% and 0.88%, respectively (Fig 1A).

In our study, occupations with high levels of exposure to live poultry, including live poultry market workers, poultry farmers and backyard farmers, showed higher infection rates. Live poultry consumption and backyard farming, which are typical in China, accelerate the spread of avian influenza virus and increase the incidence of human infection. Live poultry consumption greatly enhances the risk of human infection with avian influenza viruses and has been confirmed during the response of avian influenza H7N9 outbreak in China. Temporarily closing live poultry markets has been effective for reducing the risk of H7N9 infection in humans [25]. In addition, the lowest seroprevalence was detected in wild bird habitat workers, which is consistent with previous studies showing that the H9N2 virus predominantly circulated in poultry and that its antigenicity was different between H9N2 viruses isolated from poultry and wild birds[1].

### Seroprevalence of H9N2 viruses among different gender and age groups

The seroprevalence of both H9N2 strains was similar between gender groups for both A/Guangzhou/333/99 (G9) (2.83% in females and 3.24% in males) and A/quail/Hong Kong/G1/97 (G1) (1.05% in females and 1.3% in males) viruses (*p>*0.05) (Fig 1B). However, significant differences were observed in the seroprevalence among age groups for both H9N2 viruses using the chi-square test (*p*<0.0001), with the highest rate found in the elderly (4.45% for A/Guangzhou/333/99 (G9) and 2.31% for A/quail/Hong Kong/G1/97 (G1)), followed by adults (3.06% for A/Guangzhou/333/99 (G9) and 1.1% for A/quail/Hong Kong/G1/97 (G1)) and youths (1.38% for A/Guangzhou/333/99 (G9) and 0.39% for A/quail/Hong Kong/G1 (G1)) (Fig 1C). In children, only one serum sample presented neutralization antibodies against A/quail/Hong Kong/G1/97 (G1) in a total of 65 samples.

In our study, the seroprevalence was highest in the elderly and lowest in children. This is inconsistent with our case surveillance result. Over the past few years, most of the human cases were in children. The symptoms of human infection with H9N2 virus are very mild, whereas adults with infections who have very mild symptoms may not go to sentinel hospitals, resulting in those cases being missed in the surveillance. In this circumstance, serological investigations in large populations will present a more accurate human infection rate. In mainland China, the H9N2 virus was first reported in chickens in 1994 [26], and the first human case of H9N2 virus was reported in 1998 [6]. The H9N2 virus has been circulating in China for over twenty years, and it has become one the most predominate viruses in poultry [1, 2, 7]. The longer exposure periods for the elderly significantly increase the risk of infection and result in higher seropositivity.

### Varied geographical distribution of H9N2 viruses

In this study, serum samples were collected from 22 provinces, including 11 in Southern China and 11 in Northern China. Positive antibodies against A/Guangzhou/333/99 (G9) (S1 Table) and A/quail/Hong Kong/G1/97 (G1) (S2 Table) were detected in the serum samples collected from 21 provinces except Heilongjiang Province (n = 280), which is located in Northeast China. The seroprevalences of A/Guangzhou/333/99(G9) and A/quail/Hong Kong/G1/97 (G1) in Southern China were 3.42% and 1.37%, respectively, and were significantly higher than the seroprevalences in Northern China (2.34% for A/Guangzhou/333/99 (G9) and 0.81% for A/quail/Hong Kong/G1/97 (G1)) (*p*<0.001) (Fig 1C).

From a geographic perspective, the H9N2 virus has been widely distributed in poultry in China [1, 27], and human subclinical infection occurs widely. In only one province was there zero detection of positive serum against H9N2 virus in a total of 280 sera samples. The findings of this study that the seroprevalence of H9N2 in Southern China is much higher than that in Northern China, indicates a higher risk of infection with H9N2 in occupationally exposed populations in Southern China. Southern China, which has a higher density of live poultry sales and poultry farming compared with Northern China, is the reservoir for avian influenza viruses. Human infection with avian influenza viruses, such as H9N2 [6], H5N1 [28], H7N9 [12], H10N8 [13] and H5N6 [29], were all first identified in Southern China. Surveillance and risk assessment of avian influenza activity in Southern China have been scaled up.

## Conclusions

Sustained circulation of H9N2 virus in poultry has caused human infections. Most of these infections are mild or subclinical and are thus difficult to detect through influenza-like illness surveillance. The intensity of the population’s exposure to live poultry is the main factor underlying the higher seroprevalence detected in the elderly, those involved in live poultry sales and farming, and occupationally exposed groups in Southern China. The surveillance of H9N2 in poultry and occupationally exposed populations should be intensified, especially in Southern China.

## Supporting information

S1 Table Geographical analysis of the seroprevalence of avian influenza virus A/Guangzhou/333/99 in China. (DOC)

S2 Table Geographical analysis of the seroprevalence of avian influenza virus A/quail/Hong Kong/G1/97 in China. (DOC)
