# Supplementary material for: A comprehensive retrospective study of the seroprevalence of H9N2 avian influenza viruses in occupationally exposed populations in China
Source: PLoS One. 2017 Jun 2;12(6):e0178328. doi: 10.1371/journal.pone.0178328 (PMC5456037; doi:10.1371/journal.pone.0178328)
Supplement: S1 Table — (DOC) [file pone.0178328.s001.doc]

S1 table. Geographical analysis of the seroprevalence of avian influenza virus A/Guangzhou/333/99 in China.

| South | Tested Sera | Positive number | Seroprevalence (%) |
| --- | --- | --- | --- |
| Anhui | 825 | 25 | 3.03 |
| Fujian | 1534 | 70 | 4.56 |
| Guangdong | 1541 | 66 | 4.28 |
| Guangxi | 846 | 20 | 2.36 |
| Hunan | 2255 | 50 | 2.22 |
| Jiangsu | 407 | 7 | 1.72 |
| Jiangxi | 1046 | 36 | 3.44 |
| Shanghai | 973 | 46 | 4.73 |
| Zhejiang | 330 | 12 | 3.64 |
| Chongqing | 247 | 16 | 6.48 |
| Sichuan | 399 | 9 | 2.26 |
| North |  |  |  |
| Henan | 321 | 7 | 2.18 |
| Jilin | 629 | 7 | 1.11 |
| Liaoning | 710 | 18 | 2.54 |
| Ningxia | 411 | 7 | 1.70 |
| Qinghai | 222 | 4 | 1.80 |
| Shandong | 927 | 31 | 3.34 |
| Tianjin | 523 | 8 | 1.53 |
| Hebei | 134 | 3 | 2.24 |
| Shaanxi | 124 | 3 | 2.42 |
| Shanxi | 212 | 8 | 3.77 |
| Heilongjiang | 280 | 0 | 0.00 |
