# Supplementary material for: A comprehensive retrospective study of the seroprevalence of H9N2 avian influenza viruses in occupationally exposed populations in China
Source: PLoS One. 2017 Jun 2;12(6):e0178328. doi: 10.1371/journal.pone.0178328 (PMC5456037; doi:10.1371/journal.pone.0178328)
Supplement: S2 Table — (DOC) [file pone.0178328.s002.doc]

S2 table. Geographical analysis of the seroprevalence of avian influenza virus A/quail/Hong Kong/G1/97 in China

| South | Sera number | Positive number | Seroprevalence (%) |
| --- | --- | --- | --- |
| Anhui | 831 | 13 | 1.56 |
| Fujian | 855 | 11 | 1.29 |
| Guangdong | 1544 | 20 | 1.30 |
| Guangxi | 891 | 7 | 0.79 |
| Hunan | 1662 | 13 | 0.78 |
| Jiangsu | 407 | 6 | 1.47 |
| Jiangxi | 730 | 16 | 2.19 |
| Shanghai | 995 | 16 | 1.61 |
| Zhejiang | 308 | 7 | 2.27 |
| Chongqing | 249 | 8 | 3.21 |
| Sichuan | 400 | 5 | 1.25 |
| North |  |  |  |
| Henan | 321 | 1 | 0.31 |
| Jilin | 636 | 12 | 1.89 |
| Liaoning | 711 | 4 | 0.56 |
| Ningxia | 411 | 4 | 0.97 |
| Qinghai | 223 | 6 | 2.69 |
| Shandong | 963 | 5 | 0.52 |
| Tianjin | 523 | 1 | 0.19 |
| Hebei | 176 | 1 | 0.57 |
| Shaanxi | 124 | 1 | 0.81 |
| Shanxi | 213 | 2 | 0.94 |
| Heilongjiang | 280 | 0 | 0.00 |
